# A novel high-throughput immunofluorescence analysis method for quantifying dystrophin intensity in entire transverse sections of Duchenne muscular dystrophy muscle biopsy samples

**DOI:** 10.1371/journal.pone.0194540

**Published:** 2018-03-26

**Authors:** Valentina Sardone, Matthew Ellis, Silvia Torelli, Lucy Feng, Darren Chambers, Deborah Eastwood, Caroline Sewry, Rahul Phadke, Jennifer E. Morgan, Francesco Muntoni

**Affiliations:** 1 Dubowitz Neuromuscular Centre, Molecular Neurosciences Section, Developmental Neuroscience Programme, UCL Great Ormond Street Institute of Child Health, London, United Kingdom; 2 Department of Neurodegenerative Diseases, UCL Institute of Neurology, London, United Kingdom; 3 Division of Neuropathology, UCL Institute of Neurology, National Hospital for Neurology and Neurosurgery, UCLH NHS Foundation Trust, London, United Kingdom; 4 Department of Orthopaedics, Great Ormond Street Hospital, London, United Kingdom; 5 The Royal National Orthopaedic Hospital, Stanmore, United Kingdom; 6 Wolfson Centre for Inherited Neuromuscular Diseases, RJAH Orthopaedic Hospital, Oswestry, United Kingdom; 7 NIHR Great Ormond Street Hospital Biomedical Research Centre, London, United Kingdom; University of Minnesota Medical Center, UNITED STATES

## Abstract

Clinical trials using strategies aimed at inducing dystrophin expression in Duchenne muscular dystrophy (DMD) are underway or at advanced planning stage, including splice switching antisense oligonucleotides (AON), drugs to induce read-through of nonsense mutations and viral mediated gene therapy. In all these strategies, different dystrophin proteins, often internally deleted, are produced, similar to those found in patients with the milder DMD allelic variant, Becker muscular dystrophy (BMD). The primary biological endpoint of these trials is to induce functional dystrophin expression. A reliable and reproducible method for quantification of dystrophin protein expression at the sarcolemma is crucial to monitor the biochemical outcome of such treatments. We developed a new high throughput semi quantitative fluorescent immunofluorescence method for quantifying dystrophin expression in transverse sections of skeletal muscle. This technique is completely operator independent as it based on an automated scanning system and an image processing script developed with Definiens software. We applied this new acquisition-analysis method to quantify dystrophin and sarcolemma-related proteins using paediatric control muscles from cases without a neuromuscular disorder as well as DMD and BMD samples. The image analysis script was instructed to recognize myofibres immunostained for spectrin or laminin while dystrophin was quantified in each identified myofibre (from 2,000 to over 20,000 fibres, depending on the size of the biopsy). We were able to simultaneously extrapolate relevant parameters such as mean sarcolemmal dystrophin, mean spectrin and laminin intensity, fibre area and diameter. In this way we assessed dystrophin production in each muscle fibre in samples of DMD, BMD and controls. This new method allows the unbiased quantification of dystrophin in every myofibre within a transverse muscle section and will be of help for translational research projects as a biological outcome in clinical trials in DMD and BMD.

## Introduction

Duchenne muscular dystrophy (DMD, MIM 310200) is a severe form of muscular dystrophy caused by mutations in the dystrophin gene (*DMD*) that leads to a lack of dystrophin protein production, and results in muscle degeneration and premature death of the patients (reviewed in [1]*)*. This X-linked condition affects approximately 1/5,000 male births worldwide [2]. Genetic aberrations (nonsense mutations, exons duplication and exons deletions) interrupt the open reading frame and abolish dystrophin protein production. In order to induce dystrophin protein expression, many different approaches are being tested. These include: i) delivery of functional mini- and micro-dystrophins by recombinant adeno-associated viral (rAAV) vectors [3] ii) therapeutic exon skipping using antisense oligonucleotides (AON) [4]; iii) drugs to induce read through of nonsense mutations [5]; iv) stem cell-mediated approaches [6] and CRISPR-Cas9 genome editing strategies [7]. The most advanced of these approaches relates to the use of AONs which induce exon skipping and restore the transcript reading frame, disrupted in DMD cases carrying out of frame deletions. AONs eventually promote the translation of an internally deleted dystrophin protein, mimicking what occurs in the milder allelic condition Becker muscular dystrophy (BMD). This should prevent or delay muscle fibre degeneration and ameliorate disease progression [1]. As the primary biological endpoint of these DMD clinical trials is the production of functional dystrophin, it is crucial to have a reliable and reproducible method for dystrophin quantification. While a number of methodologies ranging from semi-quantitative western blot [8] to mass spectrometry [9] have been developed to accurately measure dystrophin in muscle lysates, a limitation of these techniques is that they do not allow the assessment of the proper localization of dystrophin at sarcolemma. As the main function of dystrophin is to stabilize a number of proteins of the dystrophin associated glycoprotein complex at the sarcolemma [10], it is crucial to know that dystrophin is correctly localized and consequently able to exert its mechanical reinforcement during muscle contraction [11]. Our group was the first to publish a detailed immunohistochemistry protocol for quantifying dystrophin expression [12]. This method exploited dystrophin and spectrin immunofluorescence quantification and is based on single labelling performed on serial transverse sections of a muscle biopsy. For each muscle sample, 40 regions of interest were analyzed by capturing sarcolemmal labelling membrane intensity and dystrophin quantification was expressed as an arbitrary intensity measure of dystrophin/spectrin signal ratios. We demonstrated that this analysis was reproducible across different laboratories [8] and we used it for quantifying dystrophin after AON administration in DMD patients enrolled in two clinical trials [13, 14]. However, this method is extremely time consuming and also depends on a skilled operator; for these reasons it is not suitable for analyzing large numbers of samples and is potentially prone to operator bias. Another image analysis method is based on a double labelling, where dystrophin and spectrin are simultaneously detected [15]. The authors created an image processing script in Metamorph software which exploited the spectrin signal for recognizing fibres and generated a computational mask of sarcolemma areas where dystrophin was quantified and expressed as average intensity. This method was based on confocal images acquired randomly and gave a representative quantification of dystrophin expression [15]. Another conceptually similar method, developed by Beekman and collaborators, is also based on a double labelling and confocal acquisition using Definiens software and allowed the evaluation of dystrophin expression in each individual fibre in representative confocal image series [16]. This method was a major step forward in dystrophin quantification, but also has some limitations as dystrophin could be quantified only in a subset of fibres; indeed only ~ 400 fibres on average were analyzed per section. Nevertheless, after appropriate operator training, both the Taylor and Beekman methods correlated well with the Arechavala method [8], but the concerns related to the potential bias for the selection of the regions of interest remained, especially in the context of clinical trials in which dystrophin restoration is one of the outcome measures. Herein, we have developed a new acquisition-analysis method that is able to capture all the intact fibres present in the muscle sections. This new analysis system is operator independent because it is based on an automated scanner and an image processing script. Furthermore, the computing analysis was instructed to simultaneously extrapolate multiple parameters for each individual fibre such as sarcolemmal and cytoplasmic dystrophin intensity expression, cytoplasmic and sarcolemmal spectrin or laminin intensity expression, number of fibres and area of sarcolemmal and cytoplasmic compartments. We demonstrate that this innovative technique gives reproducible results that extend the applicability and reproducibility of previous methods [12] [15] [16].

## Materials and methods

### Ethics statement

Skeletal muscle biopsies were obtained from the MRC Centre for Neuromuscular Diseases Biobank London (REC reference number: 14/SC/1128). This study was performed under approval by the NHS National Research Ethics Committee (REC reference number: 05/MRE12/32). Parents or legal guardians of children gave written informed consent.

### Muscle biopsies

Fresh muscle biopsies were placed on cork support, mounted with OCT (Agar Scientific, UK) and subsequently frozen in isopentane cooled in liquid nitrogen following standard procedures (Dubowitz V, Sewry C, Oldfors A. Muscle biopsy: a practical approach. 4th ed. Elsevier-Saunders; 2013). Open muscle biopsies from the quadriceps were obtained from molecularly confirmed patients with DMD or BMD. We also obtained control muscle biopsies from paediatric patients (age range from 3 to 13 years old as reported in Table 1) who underwent orthopaedic surgery and in whom a primary neuromuscular disease was excluded after extensive clinical and laboratory evaluation (henceforth referred to as ‘controls’). These control muscle biopsies were from quadriceps (sometimes vastus lateralis), gastrocnemius, peroneous longus, and abductor halluces/quadratus plantae muscles of the foot (Table 1) depending on the type of surgery. For some of them (control 3, 4 and 6), the size of the specimen was sufficient to generate two OCT blocks.

**Table 1:** Control, BMD and DMD muscle biopsies.

Patients	Muscle group	Age at the biopsy (years)	Dystrophin mutation	Functional motor score (HMAS)	Age at the assessment (years)
Control 1	Thigh	13	N/A	N/A	N/A
Control 2	Quadriceps	8	N/A	N/A	N/A
Control 3	Vastus lateralis	7	N/A	N/A	N/A
Control 4	Gastrocnemius	9	N/A	N/A	N/A
Control 5	Abductor hallucis/ quadratus plantae	16	N/A	N/A	N/A
Control 6	Peroneus longus	3	N/A	N/A	N/A
Control 7	Vastus lateralis	7	N/A	N/A	N/A
DMD 1	Quadriceps	4	c.583 C>T ex7	32/40	4
DMD 2	Quadriceps	8	c.9851 G>A ex 68	23/40	8
BMD 1	Quadriceps	7	Del exon 13	39/40	13
BMD 2	Vastus lateralis	3	Del exon 43–47	36/40	6

Functional motor score was assessed by performing the Hammersmith motor ability scale (HMAS) (Scott et al., 2012).

DMD, Duchenne muscular dystrophy; BMD, Becker muscular dystrophy; N/A, not applicable; NS, not specified.

### Sectioning and immunofluorescence labelling

Serial unfixed frozen transverse sections (7μm) were cut with a Leica 1850 CM cryostat (LEICA Microsystem, Milton Keynes, UK). Each slide carried two muscle sections to perform each experiment in duplicate. Slides were removed from the freezer and air dried for 20 minutes before staining.

#### Single labelling

Single immunolabelling was performed with rabbit polyclonal anti-dystrophin ab15277 (1:200, Abcam, Cambridge, UK), mouse monoclonal anti-dystrophin MANDYS106 (1:100, gift from Prof. Glen Morris and is now commercially available (MABT827, clone 2C6, Millipore), mouse monoclonal anti-spectrin (1:20, Leica Microsystem, Milton Keynes, UK) and rat monoclonal anti-laminin α-2 (4H8-2, 1:50, Enzo Life Science, Exeter, UK, subsequently referred as anti-laminin) antibodies. Muscle sections were incubated for 1 hour at room temperature (RT) with the primary antibodies followed by secondary biotinylated IgG antibodies raised against the matched species for 30 minutes (1:200; anti rabbit IgG or anti mouse IgG, GE Healthcare, Amersham Pl, UK). Sections were finally incubated for 15 minutes with 594 Alexa Fluor anti-streptavidin (1:1000, Thermo Fisher Scientific, Hemel Hempstead, UK). Between each incubation, three washes with phosphate buffered saline (PBS 1X, Sigma Aldrich, Dorset, UK) of three minutes each were performed. Sections were mounted using Hydromount (National Diagnostics, UK).

#### Double labelling

Two double labellings with two different combinations, depending on the antibody species compatibility, were performed. The first combination was rabbit anti-dystrophin ab15277 (1:200, Abcam, Cambridge, UK) coupled with mouse anti-spectrin (1:20, Novocastra, Milton Keynes, UK). The second combination used mouse anti-dystrophin MANDYS106 (1:100, gift from Prof. Glen Morris) paired with rat anti-laminin (1:50, Enzo Life Science, Exeter, UK). All the labellings were performed at RT. Muscle sections were incubated with the primary antibody combination (anti-dystrophin ab15277 and anti-spectrin) for 1 hour. After three washes with PBS sections were incubated with Alexa Fluor 488 conjugated anti-mouse IgG (1:100, Thermo Fisher Scientific, Hemel Hempstead, UK) and anti-rabbit biotinylated IgG (1:200; GE Healthcare, Amersham Pl, UK) for 30 minutes. PBS washes were performed and sections were incubated with Alexa Fluor 594 streptavidin conjugate (1:1000, Thermo Fisher Scientific, Hemel Hempstead, UK). For performing the second antibody combination (anti-dystrophin MANDYS106 and anti-laminin) muscle sections were incubated with the primary antibodies for 1 hour, then washed three times with PBS and incubated with Alexa Fluor 488 conjugated anti-rat IgG (1:100, Thermo Fisher Scientific, Hemel Hempstead, UK) and anti-mouse biotinylated IgG (1:200; GE Healthcare, Amersham Pl, UK) for 30 minutes. PBS washes were performed and sections were incubated for 15 minutes with Alexa Fluor 594 streptavidin conjugate (1:1000, Thermo Fisher Scientific, Hemel Hempstead, UK). Sections were mounted using Hydromount (National Diagnostic, UK). Further details of antibodies and a schematic staining procedure are given in Table 2.

**Table 2:** Antibodies used on muscle sections.

Double-staining combinations			
	Anti-dystrophin ab15277 and spectrin	Anti-dystrophin MANDYS106 and laminin	Incubation time
**Dystrophin primary antibody**	Rabbit polyclonal IgG (ab15277Abcam) Exon 77 1:200	Mouse monoclonal IgG2ak MANDYS106 (gift from Prof. Morris) Exon 43 1:100	1 hour
**Fibre identification primary antibody**	Mouse spectrin IgG2b (Novocastra NCL-SPEC1) 1:20	Rat laminin-2 (α-2-chain) IgG1 (4H8-2) (Enzo Life Science ALX-804-190-C100) 1:50	
**Secondary antibody**	Donkey anti-rabbit biotinylated IgG (GE Healthcare RPN1004V) 1:200	Sheep anti-mouse biotinylated IgG (GE Healthcare RPN1001V) 1:200	30 minutes
	Donkey anti-mouse IgG AlexaFluor 488 (Life Technologies A21202) 1:100	Donkey anti-rat IgG AlexaFluor 488 (Life Technologies A21208) 1:100	
	AlexaFluor594 Streptavidin (Life Technologies S11227) 1:1000	AlexaFluor594 Streptavidin (Life Technologies S11227) 1:1000	15 minutes

### Image acquisition

Images from serial sections cut and labelled at the same time were acquired with two different protocols. The first protocol (fluorescent microscope) based on [12], allowed the capturing of only a small area, while by performing the second protocol, using a slide scanner (Axio Scan.Z1 slides scanner), the entire section was captured.

#### Fluorescent microscope

Immunolabelled sections were acquired using a Leica DMR microscope (LEICA Microsystem, Milton Keynes, UK) interfaced to Metamorph (Molecular Devices, Downingtown, PA, USA). Images were acquired using the 12-bit Photometrics CoolSnapHQ2 camera (LEICA Microsystems, Milton Keynes, UK) which has a dynamic range between 0 and 4095 intensity units and images were acquired below the saturation limits. The acquisition procedure followed the previously published protocol [12] where excitation times for each fluorophore were established on the muscle sections obtained from two paediatric controls. The average of the controls’ exposure times was used to acquire each patient section. Four different images were acquired for each sample and subsequently analysed with the Metamorph software.

#### Axio Scan.Z1 slides scanner

Sections were scanned by the Axio Scan.Z1 slide scanner (Zeiss, Germany) equipped with a Colibri illumination system and an Orca Flash 4.0 V2 camera. For each fluorophore, exposure settings were established on sections of two paediatric controls and the average value was used to acquire patients’ sections. Scanned images were stored on a fileserver viewed and managed on the SlidePath Digital Image Hub (LEICA Microsystem, Milton Keynes, UK). Analysis was carried out with an in house script developed in Definiens Architect software (version 6.0, Definiens, Munich, Germany).

### Image processing

#### Metamorph software

From each image acquired with Metamorph software interface, 10 different regions of interest (ROI) were randomly selected and intensity measurements were assessed resulting in a total of 40 fibres analysed per sample. ROI intensity measurements included portion of the sarcolemma (maximum intensity) and portion of the cytoplasm (minimum intensity).

#### Definiens in house script

**Fig 1:**
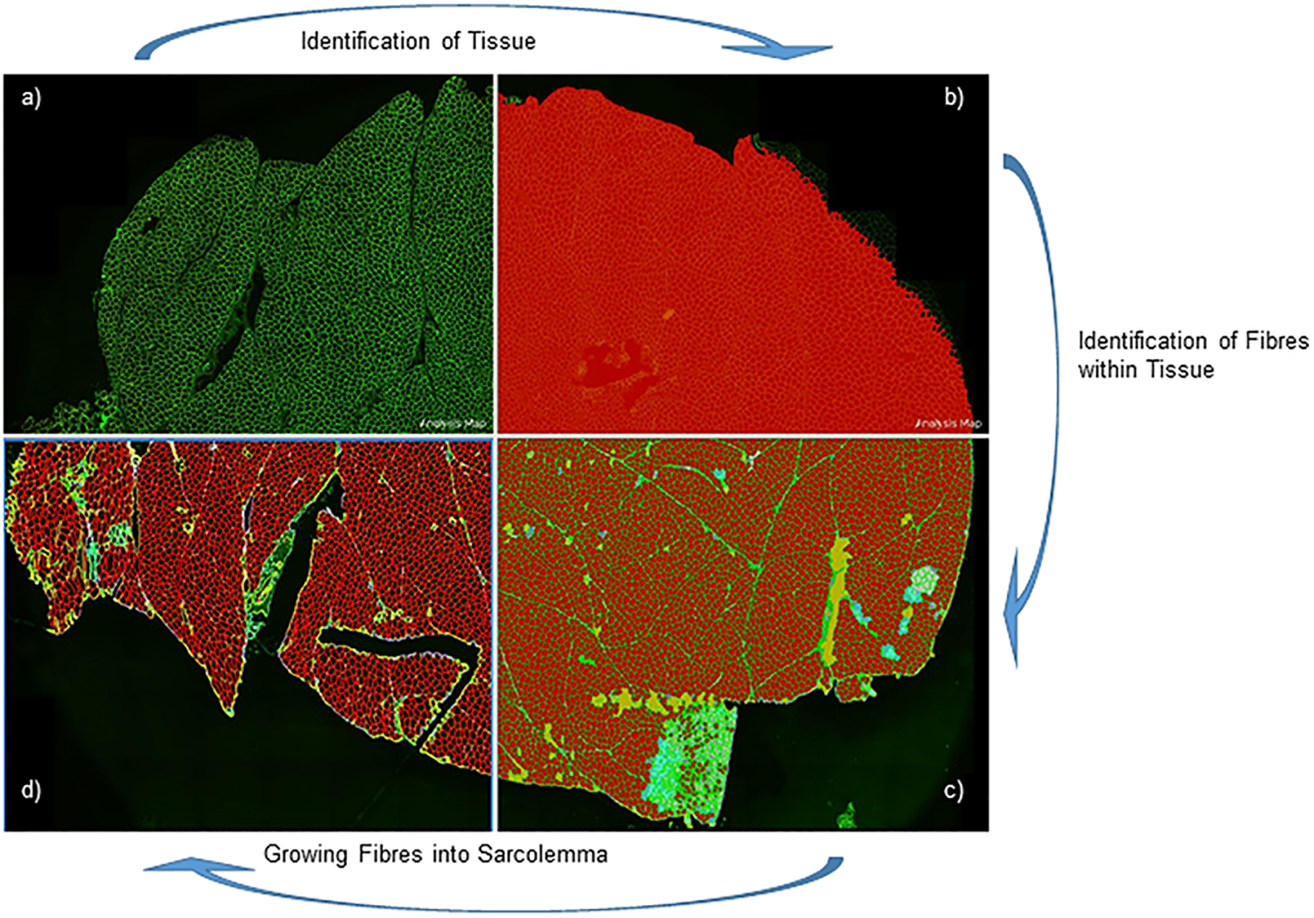
Algorithm development for identifying myofibres. Spectrin or laminin allow fibre identification within the muscle section. The algorithm was instructed to recognize the tissue within the image (a) and to subsequently recognize the myofibres within the section (b). The algorithm then generated a mask considering only structures presenting fibre characteristics (i.e. excluding nerves, spindles, blood cells, folded muscle tissue and connective areas (c). The mask generated by the algorithm included only the sarcolemma compartment of myofibres and clearly defined fibre rims (d).

Entire muscle sections were acquired by the Axio Scan.Z1 slide scanner and images were processed in Definens Architect software. We developed a customised algorithm able to simultaneously extract multiple parameters such as dystrophin intensity, fibre diameter and fibre size. The algorithm was instructed to only recognize extrafusal myofibres exploiting the spectrin or laminin labelling and created a digital mask where dystrophin was subsequently quantified in each individual myofibre (Fig 1). Depending on the size of the muscle biopsy and the resulting cross sectional area, the script was able to identify from 1,000 to over 20,000 fibres within one section. Few muscle fibres had disruptions in either spectrin or laminin staining. The algorithm was instructed to reconstruct fibre sarcolemma if the disruption was less than 20% of the entire fibre rim. We thus excluded from our analysis fibres missing more than 20% of the fibre rim, assumining that such fibres were either severely damaged or degenerating. Dystrophin mean intensity was assessed by the script using either the dystrophin (ab15277 antibody)/spectrin combination, or the dystrophin (MANDYS106 antibody)/laminin combination. Dystrophin signal was expressed in arbitrary units (au) and plotted as absolute fibre count (%) or as cumulative fibre count (%).

The time taken to perform the analysis of each section is dependent on the size of the muscle section and the corresponding size of the image files. Image acquisition takes 15–30 minutes and data acquisition 30 minutes- 1 hour/section (i.e. a total of 45 minutes– 1 hour 30 minutes/section). But these processes are automated and operator independent, guaranteeing an unbiased data set. The operator time is mainly taken up by the analysis of the raw data extrapolated by the Definiens algorithm.

### Staining reproducibility and stability

In order to assess the reproducibility of the technique and protein stability, we assessed dystrophin expression within the same patient’s muscle biopsy in two separate experiments. We used muscle blocks of two controls (control 1 and 2, Table 1). Two sets of serial transverse sections were cut and labelled. The first set of labelled sections was acquired immediately. The second set was stored at 4°C and images were acquired after three months to evaluate if the dystrophin fluorescent signal could decay across time.

### Data analysis

Scatter dot plots represented data dispersion for dystrophin quantification. The mean ± standard error of the mean (SEM) is represented for each normally-distributed data. A t-test or one way ANOVA was used to determine differences between groups. The Mann Whitney test was used for post hoc comparison. Statistical analysis was performed using GraphPad Prism version 6.07 (GraphPad Software, La Jolla, CA) and significance levels were set as: *, p<0.05; **, p<0.01 and ***, p< 0.001. The coefficient of variation (CV) was calculated to assess biological variability wherever indicated. CV was calculated using the formula CV = (standard deviation (DS) / mean) X 100.

## Results

### Development of a new high-throughput semi-automated acquisition method for identifying all muscle fibres within a transverse section

**Fig 2:**
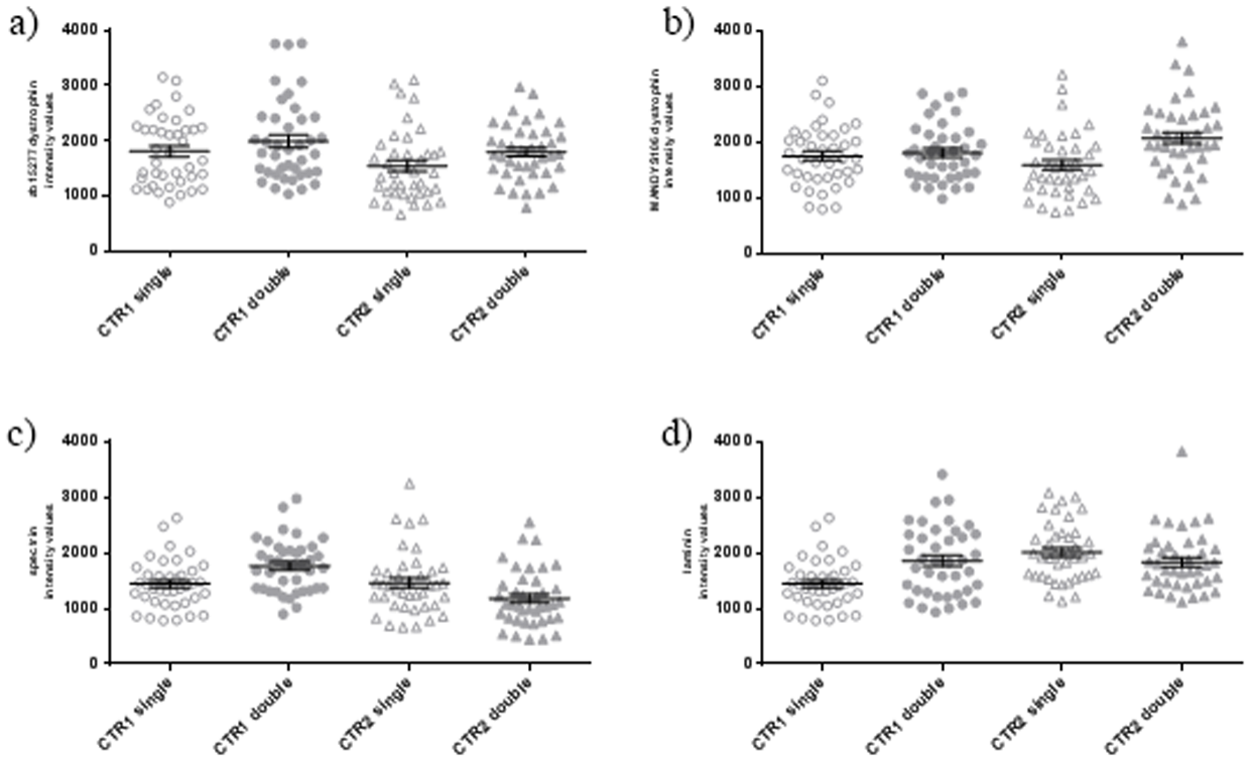
Dystrophin and sarcolemma proteins intensities used in single and double labelling. Serial sections obtained from two paediatric control muscles (control 1 and 2, Table 1) were used in single or double labelling. (Single labelling: anti dystrophin ab15277, or anti dystrophin MANDYS106; double labelling: anti dystrophin ab15277 combined with anti spectrin, or anti dystrophin MANDYS106 combined with anti laminin). For each staining, two sections were stained simultaneously and four images were acquired per section using a fluorescent microscope and analysed by Metamorph software following the Arechavala et al., 2010 method. Sarcolemma intensity values were plotted on the y axis (Mean±SEM). Dystrophin intensity values plotted as dots are the difference between dystrophin intensity at the sarcolemma and dystrophin intensity within the cytoplasm (considered to be background staining signal). Analyses were performed using anti-dystrophin ab15277 (a), anti-dystrophin MANDYS106 (b), anti-spectrin (c) and anti-laminin (d).

The customised algorithm developed in Definiens Architect software allowed us to recognize the majority of myofibres (99.8%) present within a muscle section and to calculate dystrophin mean intensity for each individual fibre. This algorithm recognized only extrafusal muscle fibres and excluded other structures present in the muscle section (such as fibrotic or necrotic areas, blood cells, vessels, nerves, spindles, adipose and connective tissue). During the algorithm development, a visual inspection of unmodified digital images was performed by one operator (M.E.) to check that the script was able to correctly identify the total number of myofibres present within the muscle section. Two different dystrophin antibodies were studied in parallel experiments: anti-dystrophin ab15277 which recognises the C-terminus of the protein and specifically an epitope in exon 77 and anti-dystrophin MANDYS106, that recognizes the protein epitope encoded by exon 43. Dystrophin quantification required myofibre identification using either spectrin when dystrophin ab15277 antibody was used, or laminin for anti-dystrophin MANDYS106. The algorithm development is summarised in Fig 1 and includes the following steps: 1) spectrin or laminin staining used independently to identify structures within the section (Fig 1a); 2) after structure identification, the algorithm generates a mask that considers only structures with myofibre characteristics (Fig 1b and 1c) (methodological paper under preparation, Ellis M. et al., 2017); 3) after fibre identification, the algorithm specifically recognised the sarcolemma compartment and clearly defined fibre peripheries (Fig 1d).

The most crucial step in the algorithm was a precise fibre identification step. To do this, we performed double labellings with either anti-spectrin or anti-laminin antibodies raised in different species compatible with the anti-dystrophin antibodies. Spectrin is generally used to assess sarcolemma integrity since it is located just under the sarcolemma while laminin is found in the basal lamina, outside the sarcolemma. Moreover, since the dystrophic pathology can disrupt the sarcolemma and potentially affect the fibre recognition step, DMD muscle sections were analysed and more than 1,000 fibres/section visually inspected revealing that less than 0.99% of fibres had interruptions in either the spectrin or laminin labelling. We found no instances of the same fibre having both laminin and spectrin staining disrupted.

### Spectrin, laminin and dystrophin mean intensity quantification with single or double labelling in control muscles

To determine if each anti-dystrophin antibody could be used in combination with spectrin or laminin without affecting the dystrophin signal intensity, we analysed mean dystrophin intensity values obtained by single anti-dystrophin labelling compared to values recorded after double labelling in serial muscle sections from two paediatric controls (control 1 and 2, Table 2). Images of labelled sections were acquired and then analysed by Metamorph following the method previously developed by us [12]. We found no significant variation in the mean dystrophin intensity when the labelling was performed using anti-dystrophin ab15277 alone or in combination with anti-spectrin (Fig 2a). Similarly, when anti-dystrophin MANDYS106 was used, there was no significant variation in the dystrophin mean intensity when the antibody was used alone or in combination with anti-laminin (Fig 2b). Moreover, we found that spectrin and laminin signals had no variation in the mean intensity when the antibodies were used either alone, or in combination with the species compatible anti-dystrophin antibody (Fig 2c and 2d).

### Mean spectrin and laminin intensity quantification in control muscles, in DMD and BMD muscle samples

**Fig 3:**
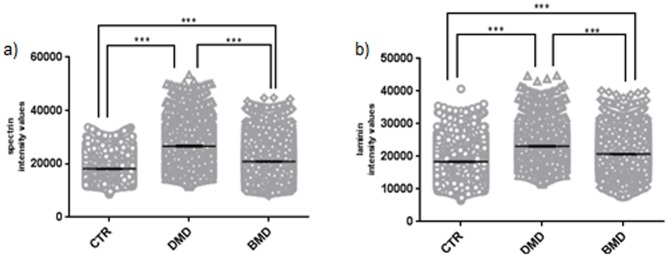
Spectrin and laminin mean intensity in muscle sections of DMD, BMD and control patients. Muscle sections were cut from two paediatric controls, two DMD and two BMD patients. Two biological replicate experiments were performed and for each experiment two sections per sample were stained with anti-spectrin or anti-laminin and acquired by the Axio Scan slide scanner. The Definens script extrapolated spectrin or laminin intensities for each individual myofibre identified within the section. Protein intensities data for spectrin (a) or for laminin (b) were grouped per category (CTR, DMD and BMD) as mean±SEM. The Mann Whitney test revealed significant differences between CTR, DMD and BMD for both spectrin (a) and laminin (b) fluorescent intensities (***, p<0.001). (CTR: control; DMD: Duchenne muscular dystrophy; BMD: Becker muscular dystrophy; SEM: standard error of the mean).

In the recent literature, dystrophin has been presented as normalised values to the sarcolemmal marker protein, spectrin [8, 12, 15]. We acquired entire muscle sections using the Axio Scan slide scanner and analysed images with the Definiens script. We found significantly higher spectrin levels, 2.65 and 1.64 fold respectively (p< 0.01) in the muscle sections of DMD and BMD patients (Table 3) compared to the average spectrin values detected in the paediatric control muscles (Fig 3a). We also found that there was a statistically significant (p< 0.01) higher level of the laminin mean intensity in the DMD and BMD muscle samples of 1.25 and 1.12 fold respectively compared to the average laminin signal detected in the control muscle sections (Fig 3b). These data suggest that using spectrin or laminin to normalise dystrophin intensity values will lead to an underestimation of the levels of dystrophin at the sarcolemma in dystrophinopathies.

**Table 3:** Labelling variability in different biological replicates of controls.

**Anti-dystrophin ab15277**										
	**Control 1**					**Control 2**				
	Nr. fibres	Max MD	Min MD	Ave MD	%CV	Nr. fibres	Max MD	Min MD	Ave MD	%CV
**EXP.A**	14559	40968	3879	18109	19	16234	36567	4729	18700	15
**EXP.B**	12447	49339	7287	23271	25	13212	41870	7238	16779	24
**EXP.C**	12924	8627	2148	4628	11	12016	22011	3769	10381	18
**Anti-dystrophin MANDYS106**										
	**Control 1**					**Control 2**				
	Nr. fibres	Max MD	Min MD	Ave MD	%CV	Nr. fibres	Max MD	Min MD	Ave MD	%CV
**EXP.A**	13245	31749	3248	10572	29	15159	28271	5962	15185	17
**EXP.B**	9961	29958	7707	17049	16	13321	37643	4614	20892	15
**EXP.C**	13530	5991	1785	3226	13	14878	16242	3342	6104	18

Muscle sections were cut from two muscle biopsies of non-neuromuscular donors and immediately stained and acquired (experiment A and experiment B). Sections were cut, stained and stored for three months and subsequently acquired in experiment C. Max MD, Maximum Mean Dystrophin; Min MD, Minimum Mean Dystrophy; Ave MD, Average Mean Dystrophin; CV, Coefficent Variation.

### Dystrophin expression in control muscles varies between different muscle types

**Fig 4:**
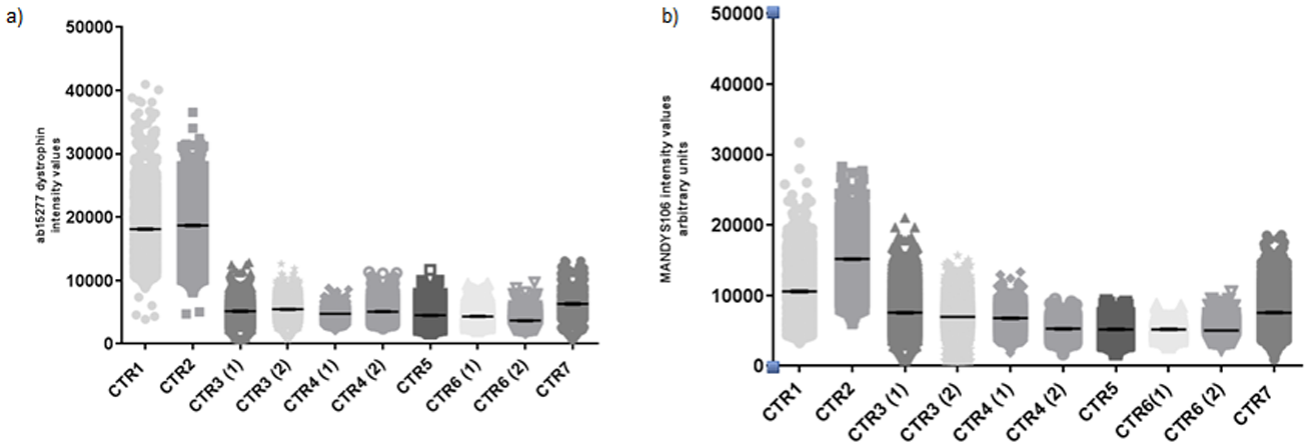
Dystrophin expression in different muscle types obtained from paediatric controls. Ten muscle blocks derived from different muscle groups obtained from seven controls (Table 1) were analysed performing anti-dystrophin ab15277/anti-spectrin and anti-dystrophin MANDYS106/anti-laminin stainings. Entire muscle sections were acquired by the Axio Scan slide scanner and processed by the Definiens algorithm, exploiting spectrin or laminin staining for fibre identification. Dystrophin was quantified in each individual fibre using either anti-dystrophin ab15277 (Fig 4a) or MANDYS106 (Fig 4b) intensities plotted as arbitrary units. We acquired a different number of myofibres (22,000 fibres for control 3 to 5,000 fibres for control 5) per section depending on the cross sectional area.

We performed the following double labellings on sections from the seven paediatric controls: anti-dystrophin ab15277 paired with anti spectrin, or anti-dystrophin MANDYS106 coupled with anti-laminin. Mean dystrophin intensity for each individual fibre identified in each muscle section was quantified and dystrophin mean intensity for each muscle specimen as well as the average value of the mean dystrophin intensity were calculated. Analysis of the control samples revealed no statistically significant differences between the average value of dystrophin intensity when either anti-dystrophin ab15277 or anti-dystrophin MANDYS106 was used. The dystrophin average intensity value in all the control muscle blocks analysed was 7607±25 i.v. (average ±SEM) when the staining was performed with anti-dystrophin ab15277 and 7533±9.41 i.v. when anti-dystrophin MANDYS1056 was used. When the average value of dystrophin mean intensity for each muscle sample analysed was compared, we observed variability depending on which type of muscle was analysed. Dystrophin labelling performed with both anti-dystrophin antibodies ab15277 and MANDYS106 gave the highest dystrophin average intensity in a quadriceps muscle (control 2, 18700±23 and 15185±22 i.v. respectively) while the lowest dystrophin average intensity was obtained in a peroneous longus muscle (control 6, 3670±11 and 5041±6 i.v. respectively). We found no significant variations in the mean dystrophin intensity between sections obtained from the two muscle blocks derived from the same muscle biopsy in any of these three non-neuromuscular donors (control 3, 4 and 6, Fig 4a and 4b).

### Dystrophin expression in control muscles varies between different independent experiments

**Fig 5:**
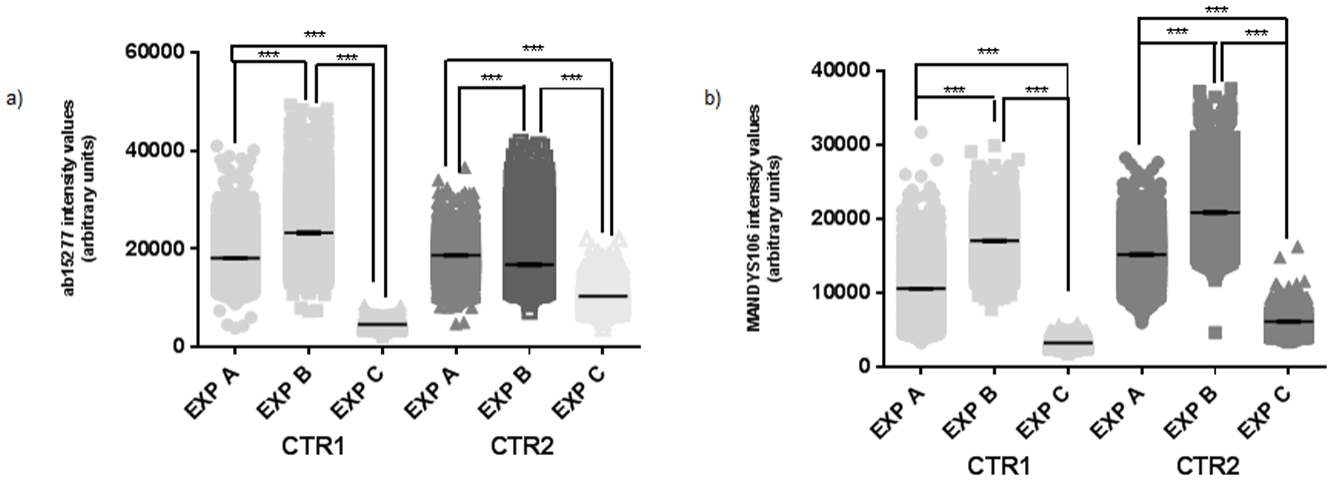
Variability in dystrophin quantification in independent immunostaining and dystrophin signal detection stability across the time. Double staining with different antibody combinations were performed (anti-dystrophin ab15277/anti spectrin (a) and anti-dystrophin MANDYS106/anti-laminin (b)). Sections were cut and stained immediately (A). Muscle blocks were kept at -80°C for one month and then two section sets were cut and again stained immediately. One set of these stained sections were acquired immediately (B) whereas the other stained section set was kept at 4°C for three months and then acquired (C) in order to evaluate the fluorescent signal stability of dystrophin staining after slides long time storage. Dystrophin intensities were quantified per each individual fibre and were plotted as scatter plots (mean±SEM) in arbitrary units (***p< 0.001, Mann Whitney test). Dystrophin intensity dynamic range and number of fibres acquired per experiment are reported in Table 3.

We then compared dystrophin expression in sections scanned immediately after staining (Fig 5, EXP A) and after the two muscle blocks were kept at -80°C for one month and sections were cut again, stained and images acquired in the same way as before (Fig 5, EXP B). There were statistically significant (p <0.01) different mean dystrophin intensity values in the two independent experiments in which muscle sections were cut, stained and acquired immediately (EXP A and B) when either anti-dystrophin ab15277 (Fig 5a) or anti-dystrophin MANDYS106 were used (Fig 5b). Coefficient variations (CV) calculated on sections obtained from two paediatric controls and stained with anti-dystrophin ab15277 varied from 19 to 25% for control 1 and from 15 to 24% for control 2 (Table 3, EXP A and B). Also by using MANDYS106 we observed variable levels between the two independent biological replicates: for control 1, we detected a %CV from 16 to 29% and from 15 to 17% for control 2. We also investigated whether the dystrophin intensity signal was affected if the image acquisition was not performed immediately after staining. To do this, sections were cut, stained and images acquired after been kept for three months at 4°C (Fig 5, EXP C). As expected after such a long storage time, a significant decrease in the dystrophin mean intensity levels was observed when the acquisition was performed three months after the staining than from serial sections in which images were acquired immediately after staining. When sections from controls were stained with ab15277, there was a reduction of 78% (for control 1) and 51% (for control 2) of dystrophin mean intensity in stained sections that had been stored for 3 months before acquisition compared to dystrophin levels observed when acquisition was performed immediately after staining (mean dystrophin value calculated by averaging EXP A and B). For sections stained with MANDYS106, there was a reduction of dystrophin mean intensity of 87% (for control 1) and 33% (for control 2) in stained sections that had been stored for 3 months before acquisition compared to the dystrophin levels obtained immediately after staining (Table 3).

### The dynamic range of dystrophin intensity and the different dystrophin average intensities when using Metamorph method

Dystrophin quantification performed by the Metamorph method [12] gave intensity values (i.v.) in a different dynamic range from the Axio Scan-Definiens method described above.

#### Anti-dystrophin ab15277

- Dynamic range

**Fig 6:**
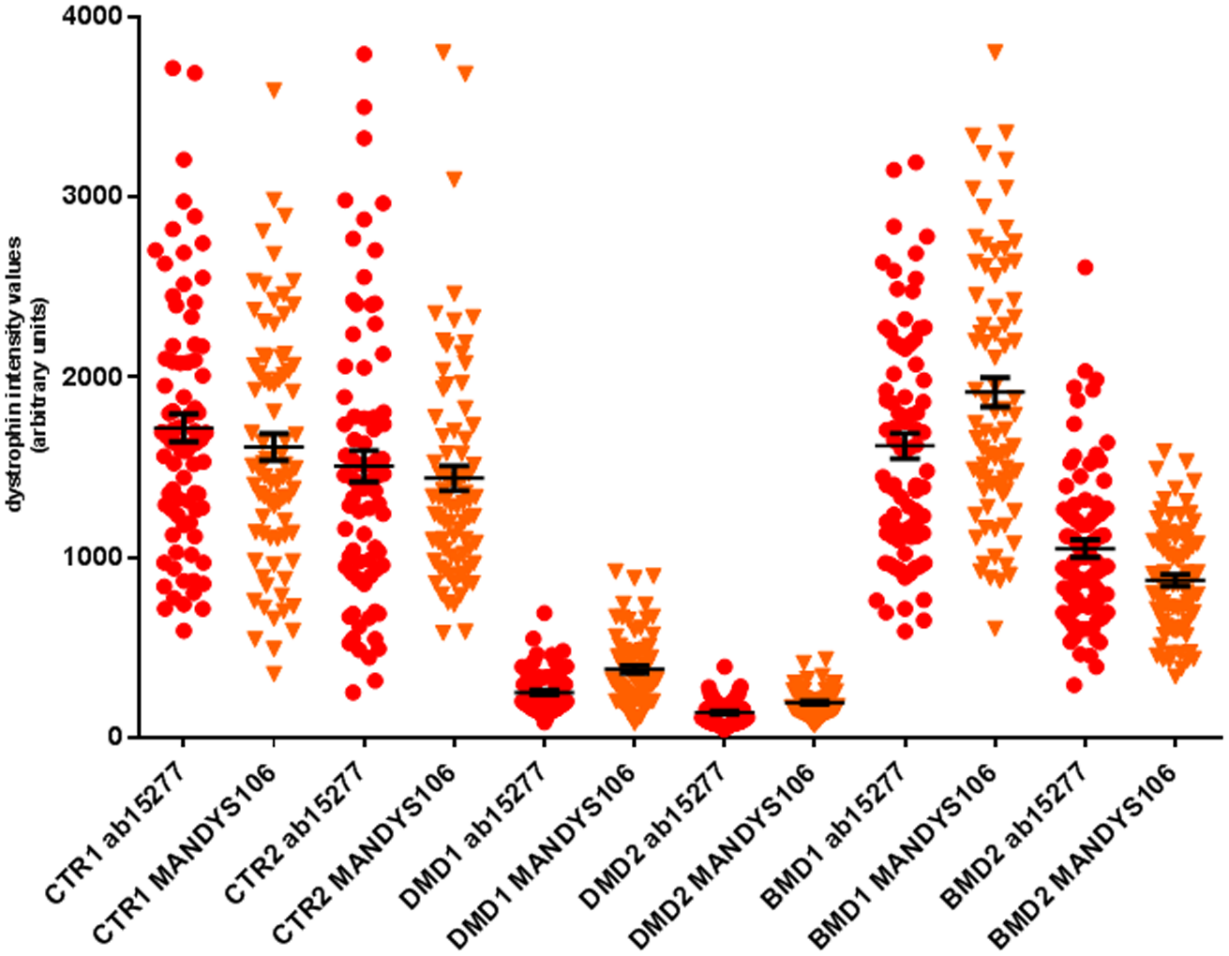
Dystrophin intensities quantification by the Arechavala et al., 2010 method using two different anti-dystrophin antibodies. Serial sections were cut from muscle blocks of two controls (control 1 and 2), two DMD and two BMD patients. Immunostainings were performed used two antibody combinations: 1) anti-dystrophin ab15277/anti spectrin and 2) anti-dystrophin MANDYS106/anti laminin. For each staining procedure two serial sections were stained as biological replicates Dystrophin intensities values recorded with anti-dystrophin ab15277 were plotted in red, whereas data obtained with MANDYS106 were displayed as orange dots. Data plots as scatter dots with mean ± SEM. CTR: control; DMD: Duchenne muscular dystrophy; BMD: Becker muscular dystrophy; SEM: standard error of the mean.

With this antibody, the highest dystrophin intensity values (i.v) for controls 1 and 2 were around 3,700, 3,193 (BMD 1), 2,611 (BMD 2), 691 (DMD 1) and 394 (DMD 2). The lowest dystrophin intensities were 590 (control 1) and 292 (control 2), 594 (BMD 1) and 252 (BMD 2), 85 (DMD 1) and 45 (DMD 2) (Fig 6).

- Average

The average dystrophin i.v. were 1,718 (control 1), 1,506 (control 2), 1,619 (BMD 1), 1,048 (BMD 2), 250 (DMD 1) and 139 (DMD 2) (Fig 6).

#### Anti-dystrophin MANDYS106

- Dynamic range

The highest dystrophin i.v. were 3,591 (control 1), 3,803 (control 2), 3,803 (BMD 1), 1,588 (BMD 2), 922 (DMD 1) and 453 (DMD 2). The lowest values were 353 (control 1), 581 (control 2), 605 (BMD 1), 339 (BMD 2), 80 (DMD 1) and 69 (DMD 2) (Fig 6).

- Average

The dystrophin intensity averages were 1,613 (control 1), 1,440 (control 2), 1,917 (BMD 1), 873 (BMD 2), 80 (DMD 1) and 69 (DMD 2), similar to the average intensities with anti-dystrophin ab15277 (Fig 6).

**Dystrophin mean intensity varies in the myofibre populations identified within the entire muscle sections of DMD and BMD patients and** controls **after Axio Scan-Definiens analysis.**

**Fig 7:**
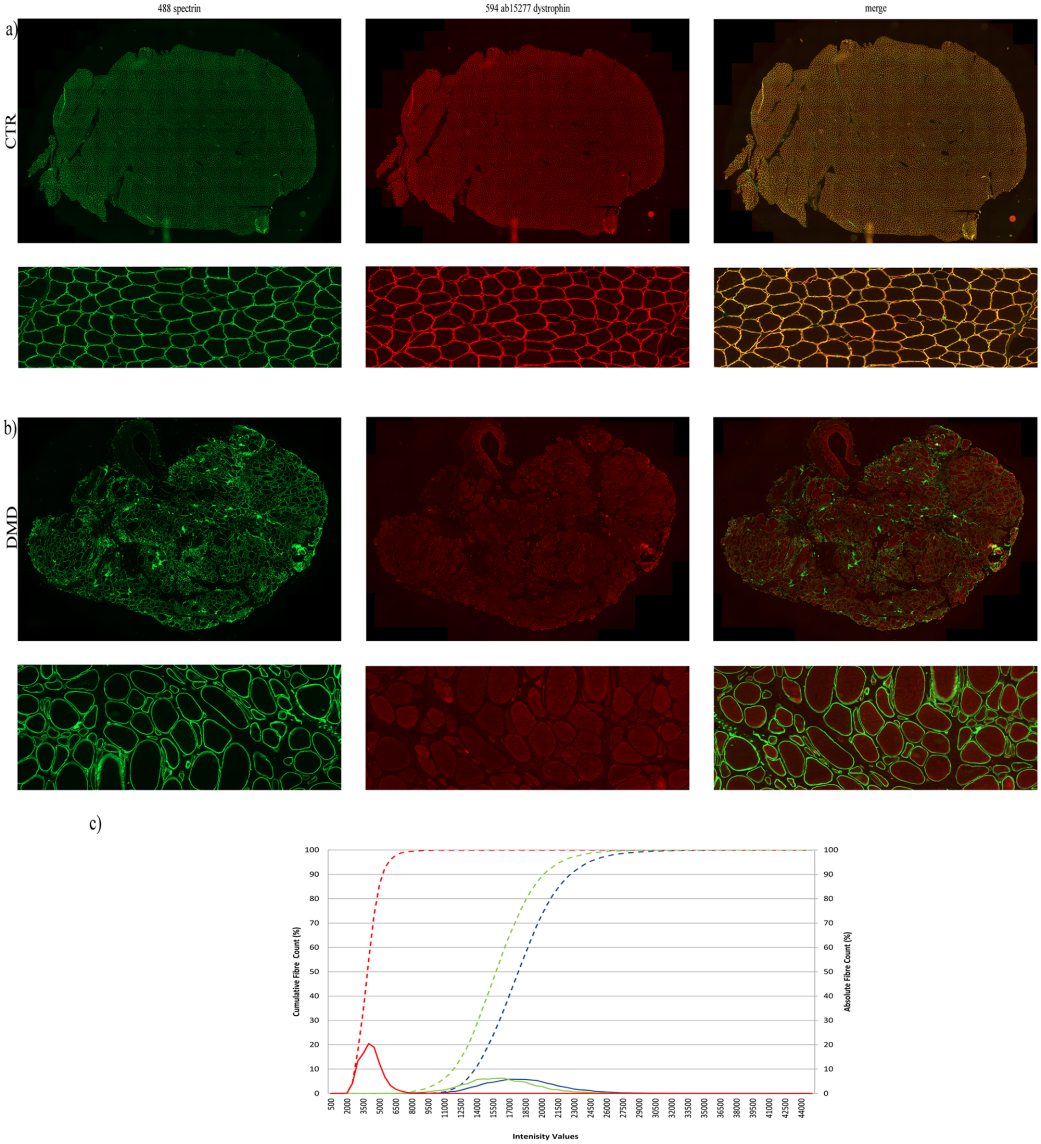
Dystrophin quantification in a population of myofibres identified in entire muscle sections performing the double labelling anti-dystrophin ab15277 and anti-spectrin. Representative images of entire muscle sections stained and acquired by the Axio Scan slide scanner and processed with Definens algorithm derived from a control (a) and from a DMD patient (b). Graph of representative dystrophin quantification in the fibre population of a control, a DMD and a BMD muscle sample (c). Dystrophin quantification was plotted as cumulative fibre count (%) on the primary y axis. The blue dashed line represents the dystrophin intensity distribution of a representative control sample (control 1, Table 1), the green dashed line represents the BMD sample and the DMD sample is represented by the red dashed line. The distribution curve of the absolute dystrophin fibre count for the control sample is represented by the blue peak, for the BMD is expressed by the green peak and the DMD fibre population curve is represented by the red peak. Fibres analysed in this representative muscle sections were: ~ 14500 for the control, ~ 10000 for the BMD and ~ 2700 for the DMD muscle section. DMD: Duchenne Muscular Dystrophy; BMD: Becker Muscular Dystrophy; i.v: intensity values.

**Fig 8:**
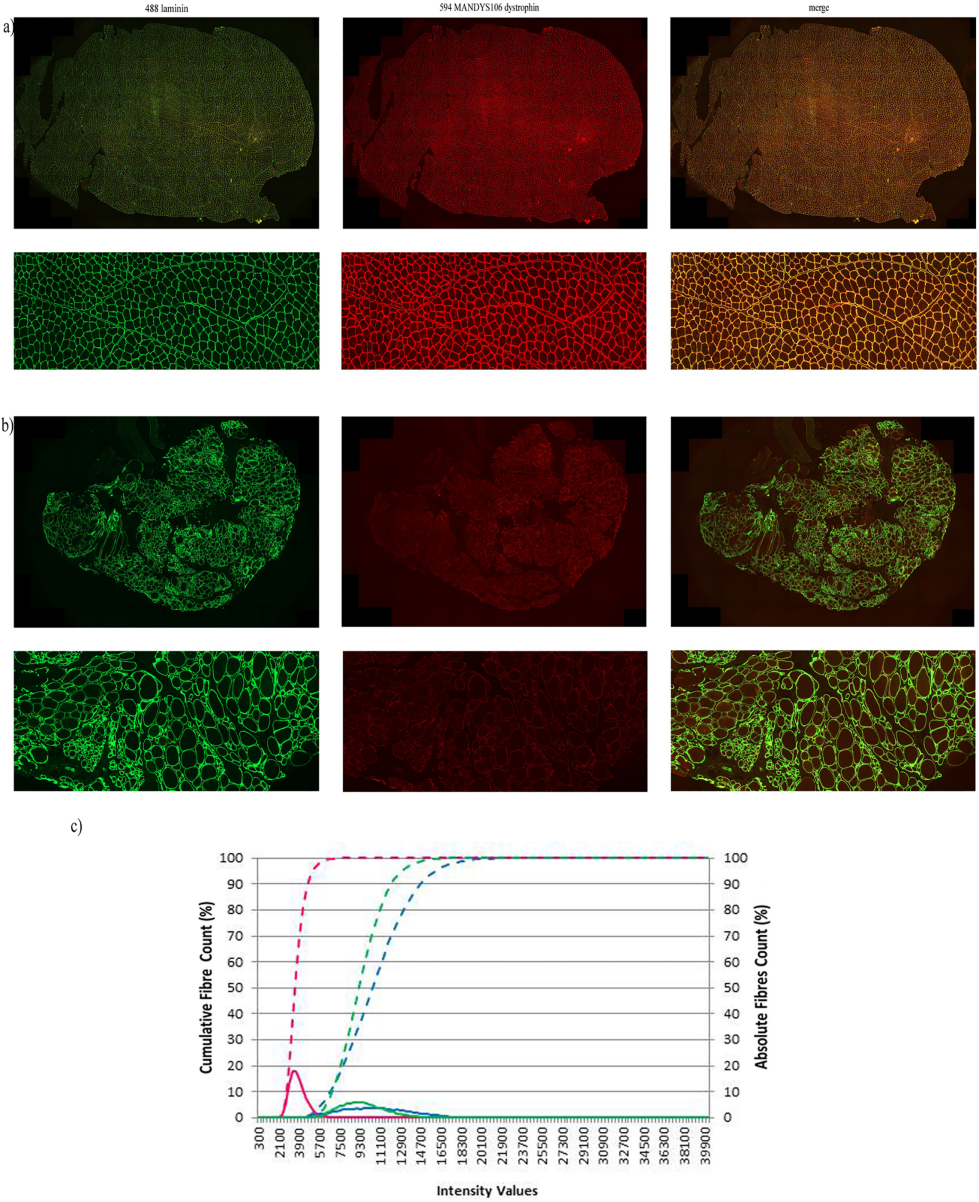
Dystrophin quantification in a population of myofibres identified in an entire muscle sections performing the double labelling anti-dystrophin MANDYS106 and anti-laminin. Representative images of entire muscle sections stained and acquired by the Axio Scan slide scanner and processed with Definiens algorithm derived from a control (a) and from a DMD patient (b). Representative dystrophin quantification in population of fibres of control, DMD and BMD muscle samples (c). Blue dashed line represents the dystrophin intensity distribution of a representative control sample (control 1, Table 1). Dystrophin absolute count obtained from the BMD sample (BMD 1, Table 1) is represented by the dark green dashed line whereas the related dystrophin intensity distribution of the DMD sample is represented by the pink dashed line. The distribution curve of the absolute dystrophin fibre count for the control sample is represented by the blue peak, for the BMD sample is expressed in dark green and the DMD sample is represented by the pink peak. Numbers of fibres analysed in these representative muscle sections were: ~ 1300 for the control, ~ 10000 for the BMD and ~ 2700 for the DMD muscle section. DMD: Duchenne Muscular Dystrophy; BMD: Becker Muscular Dystrophy; i.v: intensity values.

We observed that dystrophin is differentially expressed among individual fibres recognised within each section. There was variability in control sections as well as in both DMD and BMD patient sections, (Figs 7c and 8c) but the degree of variability was different. Dystrophin quantification data was plotted as dystrophin absolute count (%) represented by normally-distributed curves and as dystrophin cumulative fibre count (%) represented by frequency distribution curves (Figs 7c and 8c).

**Fig 9:**
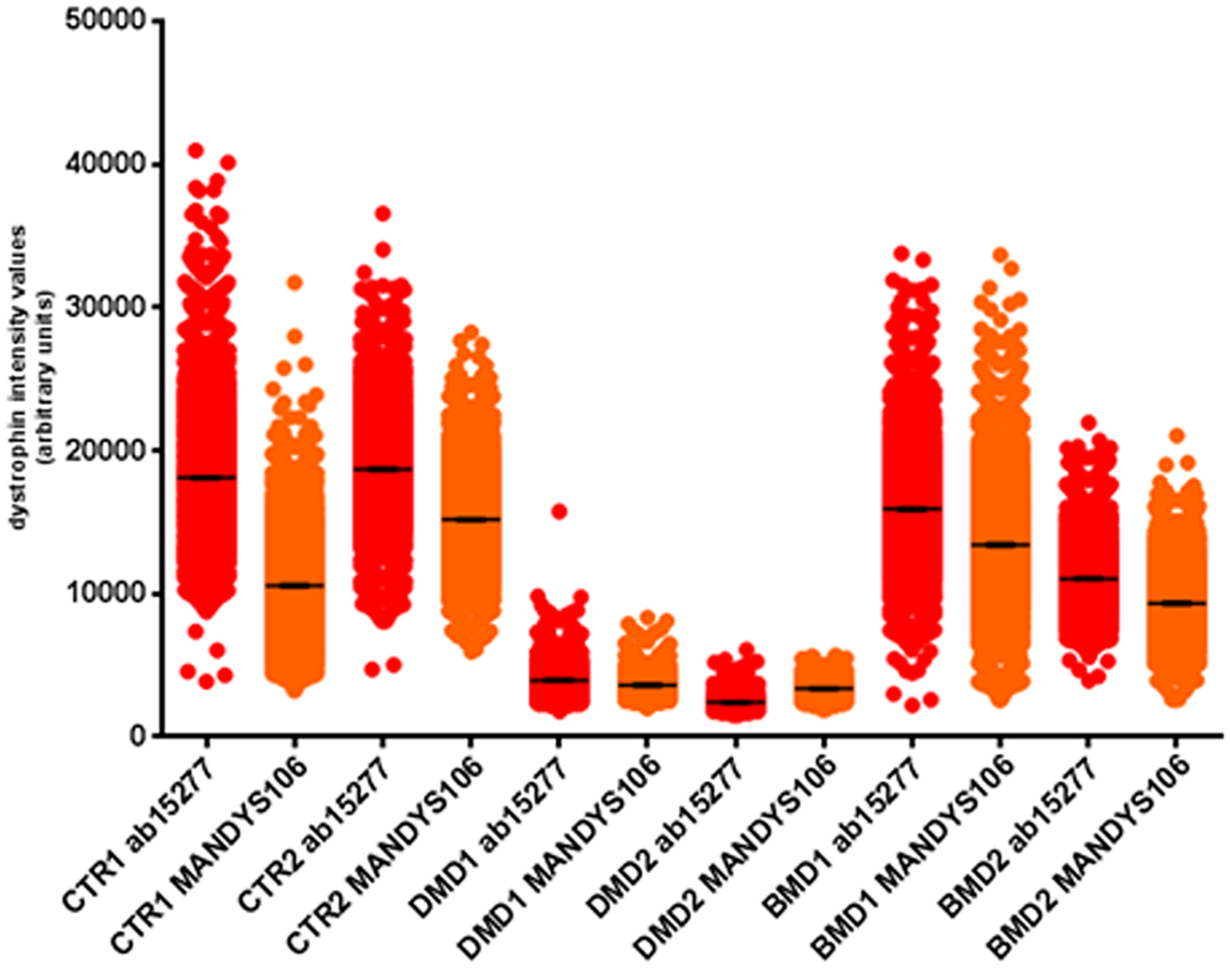
Dystrophin quantification after immunostaining with anti-dystrophin ab15277 or anti-dystrophin MANDYS106 after Axio Scan-Definiens analysis. Serial muscle sections from two controls (control 1 and 2, Table 1), two DMD and two BMD patients were cut and stained with the two antibody combinations (anti-dystrophin ab15277/anti spectrin and anti-dystrophin MANDYS106/anti-laminin). Spectrin and laminin were used only to recognize the fibres and dystrophin was quantified and expressed as absolute intensity with both ab15277 (red dots) and MANDYS106 (orange dots). The number of myofibres per sample varies and are indicated in Table 4.

- **Controls**
  Cross-sectional areas of the sections from the control muscles contained more than 10,000 fibres similar to sections of the two BMD samples (Table 4). The dynamic range of dystrophin mean intensity varied from 3879 to 49,399 i.v. when sections were stained with anti-dystrophin ab15277 (Table 4). The fibres had an average dystrophin intensity of approximately 19,000 i.v. (Table 4) and the related distribution curve of the absolute fibre count is represented by a wider peak, similar to that observed in BMD patients (Fig 7c). Control sections stained with anti-dystrophin ab15277 had a heterogeneous myofibre population expressing different levels of mean dystrophin intensity, as demonstrated by the wide dynamic range and the wide peak. Similar results were obtained using the anti-dystrophin MANDYS106 antibody (Fig 8c). Analysis of the absolute fibre count demonstrates that even for the MANDYS106 staining, control muscle sections are represented by a population of myofibres expressing different mean dystrophin intensities. After anti-dystrophin MANDYS106 staining, the myofibre population had an average dystrophin intensity of around 16,000 i.v. (Table 4) with a dynamic range of 3,248 to 37,648 i.v. (Fig 8c).
- **BMD**
  The number of myofibres (~ 10,000) present and correctly identified within BMD muscle sections after the Axio Scan-Definiens method was similar to the number of myofibres identified in the control muscle sections (Table 4). The myofibres in the BMD samples had an average dystrophin mean intensity of approximately 13,600 i.v. when anti-dystrophin ab15277 was used (Table 4). The dynamic range of dystrophin mean intensity per fibre was wider than in the DMD samples, with minimum and maximum values of 2,243 i.v. and 45,672 i.v., respectively. The broader expression is also clearly represented by a wider peak for the absolute fibre count (Fig 7c). There was a similar pattern when BMD samples were stained with MANDYS106, as the mean dystrophin intensity per fibre was represented by a broader distribution curve of the absolute fibre count (Fig 8c). Even for MANDYS106, the average of the dystrophin mean intensity in the overall myofibre population analysed in the BMD muscles is around 13,600 i.v., similar to the value obtained when ab15277 was used. The dynamic range of dystrophin mean intensity per fibre ranged from 2582 to 37,354 i.v. (Fig 9 and Table 4).
- **DMD**
  The mean dystrophin intensity using ab15277 antibody in DMD muscles varied from 1,521 to 15,756 i.v. (minimum-maximum values). Nevertheless, the fibres had mean dystrophin intensity values similar to the average value calculated for the entire population of muscle fibres (approximately 4,000 i.v.) (Table 4). Indeed, this finding is clearly illustrated by observing the absolute fibre count graph, where the myofibre population is represented by a narrow distribution curve (Fig 7c). DMD muscle sections stained with anti-dystrophin MANDYS106 gave the same pattern of variable mean dystrophin intensity values. Using this antibody, the dynamic range of dystrophin mean intensity is between 1,898 and 7,038 i.v. with the fibres expressing a mean dystrophin intensity of about 4,700 i.v. (Table 4). The absolute fibre count shows a narrow distribution curve similar to that observed using anti-dystrophin ab15277 (Fig 8c).
- **Comparison of the myofibre populations identified in the DMD/BMD and control muscle sections**
  We found that, as expected, myofibre populations in dystrophic muscles expressed a significantly lower level of dystrophin compared to paediatric control muscles. Using anti-dystrophin ab15277, we found a reduction of 83% of dystrophin mean intensity in the two DMD samples analysed compared to the dystrophin mean intensity levels in the two control muscle donors. The two BMD cases showed a modest reduction (average reduction of 29%) of dystrophin expression when BMD muscle sections were stained by anti-dystrophin ab15277 and dystrophin levels were compared to dystrophin mean expression in control sections.
  Muscle samples of the two DMD patients stained with MANDYS106 had a reduction of 70% of the mean dystrophin intensity in the control muscles. The two BMD muscle biopsies had a reduction of the mean dystrophin intensity of 14% compared to the sections of the two controls when the staining was performed using MANDYS106. Although there were no significant differences between the mean dystrophin intensity in paediatric controls compared to the BMD cases, the minimum values of the dynamic range of dystrophin detected in DMD patients muscle is similar to the minimum values in BMD sections (Table 4). The dynamic range of dystrophin mean intensity values of the control paediatric muscles is broader than in BMD muscle sections (Table 4).

**Table 4:** Intensity dystrophin range in muscle sections of DMD/BMD patients and controls.

**Dystrophin intensity ab15277**								
**Experiment 1**					**Experiment 2**			
Sample	Range values (min;MAX)	Mean±SEM	Nr.fibres/section	CV %	Range values (min;MAX)	Mean±SEM	Nr.fibres/section	CV%
Control 1	3879;40968	18109±29.58	14559	19.71	7287;49399	23271±52.66	12447	25.24
Control 2	4729;36567	18700±23.29	16234	15.86	7238;41870	16779±35.88	13212	24.58
DMD 1	1837:15756	3978±20.38	2719	26.71	3518;10140	5417±20.19	1583	20.19
DMD 2	1521;6114	2413±12.99	1557	21.24	2939;11071	4759±29.63	1112	20.76
BMD 1	2243;33781	15926±33.22	10417	21.29	2286;32372	15529±35.11	9118	21.59
BMD 2	2843;21961	11063±18.43	10857	17.36	2915;45672	12032±19.99	13267	19.14
**Dystrophin intensity MANDYS106**								
**Experiment 1**					**Experiment 2**			
Sample	Range values (min;MAX)	Mean±SEM	Nr.fibres/section	CV %	Range values (min;MAX)	Mean±SEM	Nr.fibres/section	CV%
Control 1	3248;31749	10572±26.99	13245	29.37	7707;29958	17049±27.92	9961	16.34
Control 2	5962;28271	15185±22.06	15159	22.06	4614;37648	20882±28.26	13321	15.61
DMD 1	2025;8360	3632±14.75	2732	21.23	4150;14969	7038±43.77	1466	23.81
DMD 2	1898;5703	3391±15.22	1751	18.78	4101;7624	5621±19.26	1095	11.33
BMD 1	2582;33658	13419±38.02	9820	28.08	3984;37345	15589±37.13	9358	23.03
BMD 2	2599;21047	9352±20.27	10482	22.19	6158;30687	16433±37.87	7732	20.26

DMD, Duchenne muscular dystrophy; BMD, Becker muscular dystrophy; min, minimum intensity value; MAX, maximum intensity value, SEM, standard error of the mean, %CV, % of coefficient variation.

## Discussion

The proof of concept for therapeutic approaches aimed to increase or induce dystrophin expression are currently based on dystrophin quantification performed on muscle specimens. Therefore, it is crucial to have an accurate and unbiased quantification method to monitor dystrophin expression. Herein, we have developed a high throughput, operator-independent technique to quantify dystrophin at the sarcolemma of all the myofibres present in a transverse cryosection of human skeletal muscle. To identify a myofibre, we used either spectrin or laminin staining to generate a “fibre mask” where subsequently dystrophin was quantified in each individual fibre. Spectrin and laminin are present in two different muscle compartments: spectrin is associated with the cytoskeleton of the sarcolemma, whereas laminin is a component of the extracellular membrane matrix of the basal lamina surrounding myofibres (Dubowitz V, Sewry C, Oldfors A. Muscle biopsy: a practical approach. 4th ed. Elsevier-Saunders; 2013) [17]. Nevertheless, we did not find significant differences in the number of fibres identified using either spectrin or laminin. Dystrophic muscle fibres may have interruptions at the sarcolemma and it is possible that the number of myofibres identified by spectrin or laminin could differ significantly. However, we found that in a typical DMD biopsy the number of fibres with sarcolemmal damage identified by either spectrin or laminin staining is extremely small (less than 1%). Therefore the fibre recognition step operated by either spectrin or laminin should not affect the myofibre identification and subsequent dystrophin quantification.

The results obtained with this new technique are broadly similar and in keeping with the Arechavala et al. method [12]. Nevertheless, this new method has considerable advantages over the previous one because the high-throughput acquisition facilitates the analysis of large numbers of fibres, giving a more accurate overall assessment of the dystrophin intensity in the myofibre population within an entire muscle section. As it does not require involvement of the operator in the fibre selection, potential bias is removed. The potential for bias when assessing dystrophin localisation had been recently raised by the FDA when reviewing the Sarepta filing for Eteplirsen, an antisense designed to induce exon 51 skipping (https://www.fda.gov/NewsEvents/Newsroom/PressAnnouncements/ucm521263.htm), so attempts to remove any possible operator dependent bias are necessary.

Another advantage of this technique is that dystrophin is not normalised to spectrin. This is important, as we show data (Fig 3a) indicating that spectrin levels are higher in DMD than in control muscles. A similar finding has been previously published by Beekman and collaborators who presented dystrophin data as absolute intensities, due to the higher spectrin levels observed in DMD muscles compared to control muscles [16]. We also demonstrate that spectrin levels were higher in BMD than in control muscles, although this trend for increase did not reach the statistical significance observed in DMD patients. Although the BMD samples used in this study are from very mild patients (Table 1), with high levels of residual dystrophin expression, we think it is important to highlight this finding. Importantly, these results suggest that dystrophin intensity reported in previous pre-clinical work [18, 19] and in clinical trials [13, 14] might have been underestimated, as in all these studies dystrophin intensity was normalised to spectrin intensity. Similarly, the use of spectrin to normalise utrophin and beta dystroglycan in DMD muscle samples also needs to be interpreted with this consideration [20].

Depending on the anti-dystrophin antibody used, we found different mean intensity values. Dystrophin antibody ab15277 gave slightly higher mean intensity than MANDYS106 in muscle sections of DMD/BMD patients and paediatric controls (Figs 6 and 9). This is most likely due to different affinities of the antibodies that has been observed in other studies [16]. Furthermore, ab15277 and MANDYS106 recognize different portions of the dystrophin protein. Nevertheless, using both dystrophin antibodies, we found statistically significant (p< 0.01) variability in mean dystrophin intensity in sections cut from the same control muscle biopsy but processed on different days. This is important as it provides an insight into the intrinsic variability of immunolabelling experiments and acquisition settings (Fig 3a and 3b). This was previously reported using different acquisition and analysis procedures [12, 15, 16]. There was variability in the amount of dystrophin between fibres within the same muscle section in paediatric controls as well as in DMD and BMD muscles (Figs 7c and 8c), as previously noticed by previous authors [12] [16]. The dystrophin variability is most likely due to the fact that dystrophin expression is not uniform, even in the same muscle fibre fascicle, and this could be related to the half-life of dystrophin transcript, dystrophin translation rate [10] and also epigenetic control of dystrophin production regulated by miRNA [21]. Our method analyses a high percentage (99.8%) of myofibres within the muscle, enabling us to explore for the first time the full range of dystrophin intensity variability. Our results suggest that each muscle is composed of myofibres expressing different amounts of dystrophin at the sarcolemma. Dystrophin expression has been relatively poorly studied in paediatric control muscles due to scarce availability of these samples. In a previous study performed by our group, we found no difference in dystrophin average intensity in three muscle biopsies from adolescent control muscle donors, but a fourth control muscle had significantly less dystrophin [12]. Data from another study suggests that there is a difference in dystrophin intensity between different muscle groups [16], but the numbers of muscles compared is too small to draw definite conclusions.

By using the Axio Scan/Definiens method, we found variability of mean dystrophin intensity considering the entire myofibre population within the same muscle section in all seven muscle blocks obtained from control donors. But interestingly, there was no statistically significant difference between the average dystrophin expression calculated from the seven muscle blocks and the mean intensity value generated by each muscle block analysed. This is most likely related to the large number of fibres studied, that reduces any type of data dispersion and makes average dystrophin levels homogenous in the muscles overall.

It should be noted that our method does not distinguish revertant fibres from fibres that have restored dystrophin.

There is no immunocytochemistry technique using a single dystrophin antibody that allows to recognize all revertant fibres, as different clusters of revertant fibres may express different dystrophin epitopes [22, 23]. Although revertant fibres often have high levels of dystrophin, they represent a very small percentage of the fibres in muscles of DMD patients and their numbers do not increase with time [23]. As the system we have developed allows the capture and study thousands of muscle fibres in an unbiased way, the revertant fibres are unlikely to have a significant effect on the amount of dystrophin within the entire muscle.

Another method to quantify dystrophin is Western blotting, that gives information on the amount of dystrophin and its size, but gives no information on whether this dystrophin is correctly localized at the sarcolemma (rather than being, for example, cytoplasmic). In addition, it gives no information on whether either a small proportion of fibres within a muscle has a large amount of dystrophin, or many fibres contain moderate amounts of dystrophin (both such muscles may have similar amounts of dystrophin on a blot). There is evidence from mouse studies that, for functional improvement, relatively low levels of dystrophin are required, but it must be uniformly expressed within a muscle [24] [19]. Our method allows the level of dystrophin in each fibre of a muscle biopsy to be determined and thus gives this vital biological information. This, or very similar, methods will therefore be used as a secondary endpoint, complementing Western blotting as the primary endpoint, in clinical trials designed to restore dystrophin restoration.

In conclusion, we have developed a semi-automated and high throughput method for acquiring an entire muscle section and quantifying dystrophin expression for each myofibre within that section. We demonstrate that this method is robust and operator-independent, due to the fully automated acquisition and Definiens algorithm analysis. We tested this new technique on muscle sections of paediatric control, DMD and BMD patients and we showed that the method was able to distinguish even small differences in dystrophin expression. Furthermore, we demonstrated that each dystrophin distribution curve representing either controls or DMD/BMD samples are composed by normally distributed data. Therefore, dystrophin is differentially expressed in the myofibre population within one muscle, underpinning an inherent biological variability which we describe here for the first time. The basis and consequences of this variablility are yet to be elucidated.

## References

[1] Muntoni F, Torelli S, Ferlini A. Dystrophin and mutations: one gene, several proteins, multiple phenotypes. Lancet Neurol. 2003;2(12):731–40. . 14636778 10.1016/s1474-4422(03)00585-4

[2] Straub V, Balabanov P, Bushby K, Ensini M, Goemans N, De Luca A, et al Stakeholder cooperation to overcome challenges in orphan medicine development: the example of Duchenne muscular dystrophy. Lancet Neurol. 2016;15(8):882–90. doi: 10.1016/S1474-4422(16)30035-7 . 27302365 10.1016/S1474-4422(16)30035-7

[3] Athanasopoulos T, Graham IR, Foster H, Dickson G. Recombinant adeno-associated viral (rAAV) vectors as therapeutic tools for Duchenne muscular dystrophy (DMD). Gene Ther. 2004;11 Suppl 1:S109–21. doi: 10.1038/sj.gt.3302379 . 15454965 10.1038/sj.gt.3302379

[4] Sardone V, Zhou H, Muntoni F, Ferlini A, Falzarano MS. Antisense Oligonucleotide-Based Therapy for Neuromuscular Disease. Molecules. 2017;22(4). doi: 10.3390/molecules22040563 . 28379182 10.3390/molecules22040563 PMC6154734

[5] Finkel RS, Flanigan KM, Wong B, Bonnemann C, Sampson J, Sweeney HL, et al Phase 2a study of ataluren-mediated dystrophin production in patients with nonsense mutation Duchenne muscular dystrophy. PLoS One. 2013;8(12):e81302 doi: 10.1371/journal.pone.0081302 . 24349052 10.1371/journal.pone.0081302 PMC3859499

[6] Bajek A, Porowinska D, Kloskowski T, Brzoska E, Ciemerych MA, Drewa T. Cell therapy in Duchenne muscular dystrophy treatment: clinical trials overview. Crit Rev Eukaryot Gene Expr. 2015;25(1):1–11. . 25955813 10.1615/critreveukaryotgeneexpr.2015011074

[7] Long C, Amoasii L, Mireault AA, McAnally JR, Li H, Sanchez-Ortiz E, et al Postnatal genome editing partially restores dystrophin expression in a mouse model of muscular dystrophy. Science. 2016;351(6271):400–3. doi: 10.1126/science.aad5725 . 26721683 10.1126/science.aad5725 PMC4760628

[8] Anthony K, Arechavala-Gomeza V, Taylor LE, Vulin A, Kaminoh Y, Torelli S, et al Dystrophin quantification: Biological and translational research implications. Neurology. 2014 doi: 10.1212/WNL.0000000000001025 . 25355828 10.1212/WNL.0000000000001025 PMC4248450

[9] Brown KJ, Marathi R, Fiorillo AA, Ciccimaro EF, Sharma S, Rowlands DS, et al Accurate Quantitation of Dystrophin Protein in Human Skeletal Muscle Using Mass Spectrometry. J Bioanal Biomed. 2012;Suppl 7 doi: 10.4172/1948-593X.S7-001 . 23646235 10.4172/1948-593X.S7-001 PMC3642779

[10] Fairclough RJ, Wood MJ, Davies KE. Therapy for Duchenne muscular dystrophy: renewed optimism from genetic approaches. Nat Rev Genet. 2013;14(6):373–8. doi: 10.1038/nrg3460 . 23609411 10.1038/nrg3460

[11] Petrof BJ, Shrager JB, Stedman HH, Kelly AM, Sweeney HL. Dystrophin protects the sarcolemma from stresses developed during muscle contraction. Proc Natl Acad Sci U S A. 1993;90(8):3710–4. . 8475120 10.1073/pnas.90.8.3710 PMC46371

[12] Arechavala-Gomeza V, Kinali M, Feng L, Brown SC, Sewry C, Morgan JE, et al Immunohistological intensity measurements as a tool to assess sarcolemma-associated protein expression. Neuropathol Appl Neurobiol. 2010;36(4):265–74. Epub 2009/12/17. doi: 10.1111/j.1365-2990.2009.01056.x . 20002311 10.1111/j.1365-2990.2009.01056.x

[13] Kinali M, Arechavala-Gomeza V, Feng L, Cirak S, Hunt D, Adkin C, et al Local restoration of dystrophin expression with the morpholino oligomer AVI-4658 in Duchenne muscular dystrophy: a single-blind, placebo-controlled, dose-escalation, proof-of-concept study. Lancet Neurol. 2009;8(10):918–28. doi: 10.1016/S1474-4422(09)70211-X . 19713152 10.1016/S1474-4422(09)70211-X PMC2755039

[14] Cirak S, Arechavala-Gomeza V, Guglieri M, Feng L, Torelli S, Anthony K, et al Exon skipping and dystrophin restoration in patients with Duchenne muscular dystrophy after systemic phosphorodiamidate morpholino oligomer treatment: an open-label, phase 2, dose-escalation study. Lancet. 2011 Epub 2011/07/26. doi: 10.1016/S0140-6736(11)60756-3 . 21784508 10.1016/S0140-6736(11)60756-3 PMC3156980

[15] Taylor LE, Kaminoh YJ, Rodesch CK, Flanigan KM. Quantification of dystrophin immunofluorescence in dystrophinopathy muscle specimens. Neuropathol Appl Neurobiol. 2012;38(6):591–601. doi: 10.1111/j.1365-2990.2012.01250.x . 22243335 10.1111/j.1365-2990.2012.01250.x

[16] Beekman C, Sipkens JA, Testerink J, Giannakopoulos S, Kreuger D, van Deutekom JC, et al A sensitive, reproducible and objective immunofluorescence analysis method of dystrophin in individual fibers in samples from patients with duchenne muscular dystrophy. PLoS One. 2014;9(9):e107494 doi: 10.1371/journal.pone.0107494 . 25244123 10.1371/journal.pone.0107494 PMC4171506

[17] Malandrini A, Villanova M, Sabatelli P, Squarzoni S, Six J, Toti P, et al Localization of the laminin alpha 2 chain in normal human skeletal muscle and peripheral nerve: an ultrastructural immunolabeling study. Acta Neuropathol. 1997;93(2):166–72. . 9039464 10.1007/s004010050598

[18] Anthony K, Arechavala-Gomeza V, Ricotti V, Torelli S, Feng L, Janghra N, et al Biochemical characterization of patients with in-frame or out-of-frame DMD deletions pertinent to exon 44 or 45 skipping. JAMA neurology. 2014;71(1):32–40. doi: 10.1001/jamaneurol.2013.4908 . 24217213 10.1001/jamaneurol.2013.4908

[19] Godfrey C, Muses S, McClorey G, Wells KE, Coursindel T, Terry RL, et al How much dystrophin is enough: the physiological consequences of different levels of dystrophin in the mdx mouse. Hum Mol Genet. 2015;24(15):4225–37. doi: 10.1093/hmg/ddv155 . 25935000 10.1093/hmg/ddv155 PMC4492390

[20] Janghra N, Morgan JE, Sewry CA, Wilson FX, Davies KE, Muntoni F, et al Correlation of Utrophin Levels with the Dystrophin Protein Complex and Muscle Fibre Regeneration in Duchenne and Becker Muscular Dystrophy Muscle Biopsies. PLoS One. 2016;11(3):e0150818 doi: 10.1371/journal.pone.0150818 . 26974331 10.1371/journal.pone.0150818 PMC4790853

[21] Fiorillo AA, Heier CR, Novak JS, Tully CB, Brown KJ, Uaesoontrachoon K, et al TNF-alpha-Induced microRNAs Control Dystrophin Expression in Becker Muscular Dystrophy. Cell reports. 2015;12(10):1678–90. doi: 10.1016/j.celrep.2015.07.066 . 26321630 10.1016/j.celrep.2015.07.066 PMC4757433

[22] Lu QL, Morris GE, Wilton SD, Ly T, Artem’yeva OV, Strong P, et al Massive idiosyncratic exon skipping corrects the nonsense mutation in dystrophic mouse muscle and produces functional revertant fibers by clonal expansion. J Cell Biol. 2000;148(5):985–96. . 10704448 10.1083/jcb.148.5.985 PMC2174546

[23] Arechavala-Gomeza V, Kinali M, Feng L, Guglieri M, Edge G, Main M, et al Revertant fibres and dystrophin traces in Duchenne muscular dystrophy: implication for clinical trials. Neuromuscul Disord. 2010;20(5):295–301. Epub 2010/04/17. doi: 10.1016/j.nmd.2010.03.007 . 20395141 10.1016/j.nmd.2010.03.007

[24] Sharp PS, Bye-a-Jee H, Wells DJ. Physiological characterization of muscle strength with variable levels of dystrophin restoration in mdx mice following local antisense therapy. Mol Ther. 2011;19(1):165–71. doi: 10.1038/mt.2010.213 . 20924363 10.1038/mt.2010.213 PMC3017444

